# White Matter Microstructure Associations of Cognitive and Visuomotor Control in Children: A Sensory Processing Perspective

**DOI:** 10.3389/fnint.2018.00065

**Published:** 2019-01-14

**Authors:** Annie Brandes-Aitken, Joaquin A. Anguera, Yi-Shin Chang, Carly Demopoulos, Julia P. Owen, Adam Gazzaley, Pratik Mukherjee, Elysa J. Marco

**Affiliations:** Neuroscape Center, Departments of Neurology, Pediatrics, Physiology, Radiology, and Psychiatry, University of California, San Francisco, San Francisco, CA, United States

**Keywords:** DTI, cognitive control, visuomotor control, sensory processing dysfunction, attention

## Abstract

**Objective**: Recent evidence suggests that co-occurring deficits in cognitive control and visuomotor control are common to many neurodevelopmental disorders. Specifically, children with sensory processing dysfunction (SPD), a condition characterized by sensory hyper/hypo-sensitivity, show varying degrees of overlapping attention and visuomotor challenges. In this study, we assess associations between cognitive and visuomotor control abilities among children with and without SPD. In this same context, we also examined the common and unique diffusion tensor imaging (DTI) tracts that may support the overlap of cognitive control and visuomotor control.

**Method**: We collected cognitive control and visuomotor control behavioral measures as well as DTI data in 37 children with SPD and 25 typically developing controls (TDCs). We constructed regressions to assess for associations between behavioral performance and mean fractional anisotropy (FA) in selected regions of interest (ROIs).

**Results**: We observed an association between behavioral performance on cognitive control and visuomotor control. Further, our findings indicated that FA in the anterior limb of the internal capsule (ALIC), the anterior thalamic radiation (ATR), and the superior longitudinal fasciculus (SLF) are associated with both cognitive control and visuomotor control, while FA in the superior corona radiata (SCR) uniquely correlate with cognitive control performance and FA in the posterior limb of the internal capsule (PLIC) and the cerebral peduncle (CP) tract uniquely correlate with visuomotor control performance.

**Conclusions**: These findings suggest that children who demonstrate lower cognitive control are also more likely to demonstrate lower visuomotor control, and vice-versa, regardless of clinical cohort assignment. The overlapping neural tracts, which correlate with both cognitive and visuomotor control suggest a possible common neural mechanism supporting both control-based processes.

## Introduction

Historically, individual differences in cognitive control and visuomotor control have been studied as distinct processes, however growing evidence suggests that these two domains may be interrelated (Diamond, 2000; Rasmussen and Gillberg, 2000; Brandes-Aitken et al., 2018). Here we define cognitive control as the mental processes of attention, working memory and goal management (Anguera and Gazzaley, 2015) and visuomotor control as the processes by which an individual integrates visual-perception and fine motor coordination systems (Schultz et al., 1998). Research has demonstrated that both cognitive and visuomotor control are a fundamental for academic and socioemotional development (Davidson et al., 2006; Dziuk et al., 2007; Luna et al., 2010; MacDonald et al., 2013; Sumner et al., 2016).

With respect to the domain of cognitive control, attention is an especially important process that supports adaptive development (Davidson et al., 2006; Luna et al., 2010). The ability to focus on specific stimuli while ignoring distractions over sustained periods of time is necessary to effectively process, encode and retain relevant information from the environment (Zanto et al., 2011). These processes have particularly important implications in academic and social contexts (Kenworthy et al., 2009; Stevens and Bavelier, 2012). Furthermore, goal management is the cognitive foundation for goal-directed behavior such as planning and problem solving (Levine et al., 2000; Lustig et al., 2009). Likewise, visuomotor challenges have broad implications for everyday life, from deficiencies in handwriting (Fuentes et al., 2010; Kushki et al., 2011; Johnson et al., 2013; Rosenblum et al., 2016) to academic performance and self-perception (Feder and Majnemer, 2007; Cahill, 2009).

Cognitive control and visuomotor control deficits are common to many neurodevelopmental disorders (Pennington and Ozonoff, 1996; Kaiser et al., 2015), including those who have attention deficit/hyperactivity disorder (ADHD; American Psychiatric Association. Task Force on DSM-V, 2013) and developmental coordination disorder (DCD; American Psychiatric Association. Task Force on DSM-V, 2013). A high behavioral comorbidity of attention and visuomotor control deficits exists within ADHD and DCD populations (approximately 50%; Kadesjö and Gillberg, 1999; Pitcher et al., 2003), suggesting that these processes may be interrelated (Diamond, 2000; Anguera et al., 2010; Brandes-Aitken et al., 2018). Notably, individuals with broadly-defined sensory processing dysfunction (SPD; Ahn et al., 2004; Gowen and Hamilton, 2013; Craig et al., 2016), a disorder characterized by challenges with sensory modulation, discrimination and sensory-based motor challenges (Miller et al., 2007) also show overlapping challenges related to cognitive control and visuomotor control (Ahn et al., 2004; Anguera et al., 2017; Brandes-Aitken et al., 2018). The compelling possibility exists that abnormal sensory integration accompanies deficits in both cognitive control and visuomotor control, with shared neural underpinnings given the overlapping neural architecture that connects thalamic sensory centers to frontal cortical regions (Mori et al., 2008). Indeed, previous SPD research has demonstrated decreased integrity in sensory-rich thalamocortical tracts that correlate with parent-reported inattention (Owen et al., 2013) and proprioception abilities (Chang et al., 2016).

There are a number of compelling data-driven findings that would support the aforementioned hypothesis. To begin with, a frontal cortico-striatal-thalamic network has been suggested to support behavioral-inhibitory processes, including proactive cognitive and motor processes (Jahanshahi et al., 2015). Along those same lines, the prefrontal cortex and motor cortex are anatomically adjacent and share vast reciprocal interconnections, likely contributing to the observed phenotypic overlap (Barbas and Pandya, 1987; Burman et al., 2014). The use of distinct neuroimaging techniques [electroencephalography (EEG) and functional near-infrared spectroscopy (fNIRS)] has revealed patterns of neural coherence and activity within the prefrontal cortex during cognitive-motor tasks indicative of such frontal regions facilitating visuomotor performance (Hatakenaka et al., 2007; Gentili et al., 2013, 2015).

These findings agree with structural architecture work suggesting that integrity within specific neural tracts can predict cognitive control and visuomotor control abilities. More specifically, research has found that tracts which terminate in the frontal cortex are commonly associated with attention and executive functions (Luppino et al., 1993; Ashtari et al., 2005; Pavuluri et al., 2009; de Luis-García et al., 2015; Ursache and Noble, 2016). Interestingly, these frontal-related tracts also play a role in visuomotor control (Steele et al., 2012; Langevin et al., 2014). However, it should be noted that there are a number of other regions that have been associated with supporting each process. For example, posterior parietal and cerebellar regions are neural areas specifically associated with visuomotor abilities (Martin, 2005; Paulin, 2008; Zwicker et al., 2012a; Koziol et al., 2014; Song et al., 2015) while superior parietal regions are associated with higher-order cognitive abilities (Sylvester et al., 2003; Collette et al., 2006).

Based on these previous findings, we hypothesize that specific white matter tracts, identified using diffusion tensor imaging (DTI), would correlate with either cognitive control, visuomotor control, or both. For example, given that the anterior limb of the internal capsule (ALIC), anterior thalamic radiation (ATR), and superior longitudinal fasciculus (SLF) share connections within the frontal lobe, we would expect their tract integrity to correlate with performance on both cognitive control (Luppino et al., 1993; Ashtari et al., 2005; Pavuluri et al., 2009; de Luis-García et al., 2015; Ursache and Noble, 2016) and visuomotor control (Steele et al., 2012; Langevin et al., 2014) tasks. Conversely, integrity of the superior corona radiata (SCR), which has been associated with dual-task processing (Seghete et al., 2013), is predicted to show associations with cognitive control abilities (Koenigs et al., 2009; Stave et al., 2017). Finally, the integrity of the cerebral peduncle (CP), the posterior thalamic radiation (PTR), and the posterior limb of the internal capsule (PLIC), classically found to be associated with sensory and motor information transmission (Martin, 2005; Paulin, 2008; Zwicker et al., 2012a,b; Koziol et al., 2014), is predicted to correlate only with visuomotor function.

Here, we were interested in exploring the interrelation of cognitive control and visuomotor control processes across children with SPD and typically developing controls (TDCs). Given that our previous work has already established group-based differences in attention, visuomotor control and fractional anisotropy (FA; see Owen et al., 2013; Chang et al., 2014, 2016; Anguera et al., 2017; Brandes-Aitken et al., 2018), this article aims to take a phenotypic-first approach to assess brain behavior-relations, more generally. To do so, we intentionally pooled both the SPD and TDC groups together in our analysis to optimize variable dispersion. In the current analysis, we first assessed the behavioral association between directly assessed cognitive control and visuomotor control performance. Second, on a subset of our participants that completed the neuroimaging portion of the study, we examined the neural networks associated with cognitive and visuomotor control performance using a common measure of white matter microstructure, FA. We hypothesized that: (i) cognitive control and visuomotor control performance would be associated with each other; (ii) white matter tracts that share connections with the frontal lobe would be associated with both cognitive control and visuomotor control; and (iii) white matter tracts that are primarily connected to superior parietal regions would correlate with cognitive control, whereas tracts which primarily connect the brainstem and posterior parietal regions would correlate with visuomotor control ability. Assessing control from a neural architecture vantage will clarify how and why these two control deficits often co-exist, and elucidate the underlying neural basis of these co-occurring control processes.

## Materials and Methods

### Demographics

Participants were recruited from the UCSF Sensory Neurodevelopment and Autism Program (SNAP) clinic, SNAP research registry, and local online parent groups. We recruited a total of 62 participants between 8 years and 12 years of age: 37 children with SPD (16 female; age 10 ± 1.4 years) age and gender matched with 25 TDC children (12 female; age 10.5 ± 1.3 years). All participants successfully completed the visuomotor control battery, 35 SPD and 24 TDC children completed all cognitive control tasks, and 27 SPD and 16 TDC children provided usable DTI data for the current analysis. See Table 1 for complete demographic information. This study was carried out in accordance with the recommendations of “APA Ethics Guidelines.” The protocol was approved by the “UCSF Institutional Review Board.”

**Table 1:** Demographics.

	SPD (*N* = 37)	TDC (*N* = 25)
Age, M ± SD (range)	10 ± 1.4 (8.0–12.9)	10.5 ± 1.3 (8.0–12.8)
Gender, N		
Female	16	12
Male	21	13
Handedness, N	
Right	35	24
Left	2	1
IQ, M ± SD	
NVIQ	110.8 ± 16.0	115.3 ± 11.0
VIQ	116.0 ± 14.7	123.8 ± 11.0
Ethnicity, N		
Caucasian	24	14
Asian	0	2
African American	0	2
Mixed Ethnicity	12	2
Unknown	1	5

### Screening Procedures

Inclusion criteria for the SPD cohort was based on the widely used sensory assessment, the Sensory Profile, a parent report questionnaire (Dunn, 1999). All children in the SPD cohort had a community diagnosis of SPD and a score on the Sensory Profile in the “Definite Difference” range (<2% probability) in one or more of the sensory domains (auditory, visual, oral/olfactory, tactile, vestibular, or multisensory processing). All children were administered The Social Communication Questionnaire (SCQ) to screen for any additional social communication challenge that might meet diagnostic criteria for Autism Spectrum Disorder (ASD). Any participant who scored above 15 points was administered the Autism Diagnostic Observation Schedule, Module 3 (ADOS; Lord et al., 1989) and excluded if they met ASD criteria on the ADOS. Two participants scored above SCQ threshold and were administered an ADOS in which they scored as non-spectrum and were subsequently included in our SPD group for analysis. One subject from the SPD group was excluded for scoring above 15 points on the SCQ and declining an ADOS assessment. Participants were assigned to the TDC group if they did not meet ASD cut-off on the SCQ, ADHD cut-off on the Vanderbilt (Wolraich et al., 2003) or SPD cut-off on the Sensory Profile. In addition, participants were included in the TDC cohort only if they did not have community neurological or psychiatric diagnoses.

Seven children in our SPD cohort were on medication for attention/impulsivity symptoms and/or emotional regulation, one child in each group was on medications for allergies. All children were required to be on a stable dose of prescribed medications for at least 6 weeks prior to participating. Exclusion criteria were premature delivery (<37 weeks), brain malformation or injury, movement disorder, bipolar disorder, psychotic disorder, hearing impairment, or Perceptual Reasoning Index (PRI) score <70 on the Wechsler Intelligence Scale for Children—Fourth Edition (Wechsler, 2003).

### Cognitive Control Assessments

#### Selective/Sustained Attention

##### Test of Variables of Attention (TOVA)

We administered the test of variables of attention (TOVA; Greenberg et al., 1996) to assess sustained attention in our participants. The TOVA has demonstrated an estimated 85% sensitivity as a predictor of ADHD (Schatz et al., 2001). It is a 23-min, fixed interval, visual continuous performance task administered on a laptop computer. Participants are instructed to respond to a visual stimulus (white square) appearing in the top edge (target stimuli) of the computer screen and to ignore the stimuli when it appears at the bottom edge (non-target stimuli) of the computer. The stimuli appear for 100 ms every 2 s. The assessment is broken up into two parts measuring sustained attention (target stimuli appears in 22% of trails) and impulsivity (target stimuli appears in 77% of trials). Here, we assessed response time (RT) from the sustained condition, in line with previous work using this measure in related populations (Anguera et al., 2017).

#### Goal Management

##### Project: EVO™ (EVO)—Multi

Project: EVO™ (EVO) is proprietary software developed by Akili Interactive Labs, specifically designed as a medical device to assess cognitive and visuomotor control. EVO was developed from the principles of a previous cognitive intervention known as NeuroRacer (Anguera et al., 2013) but modified for iOS mobile compatibility. The EVO assessment includes three tasks: perceptual discrimination, visuomotor tracking, and multitasking by performing each aforementioned task simultaneously (Goal Management Task).

Here, we measured performance on the goal management task. In this assessment, the player simultaneously completes a perceptual discrimination task while also performing a visuomotor tracking task. In the perceptual discrimination component of the assessment, the user taps the iPad screen for correctly colored target stimuli while ignoring distracting targets. In the visuomotor tracking component of the assessment, the player navigates their EVO character through a dynamically moving environment by moving the iPad with the goal of avoiding the walls and obstacles. EVO incorporates adaptive psychometric staircase algorithms to ensure that comparisons between individuals reflect actual differences and not testing-based disparities. EVO changes its level of difficulty in a dynamic, trial-by-trial basis until the participant is performing at ~80% rate of accuracy (Klein, 2001; Leek, 2001; García-Pérez, 2013). This approach also helps mitigate against any biases of age-related slowing, instrumentation, or ceiling/floor effects, thus finding an individualized level of performance that is specific to each child. The EVO assessment takes approximately 7 min. Here we specifically focused on performance during the multitasking condition to avoid redundancy with our other attentional measures. Here, we assessed EVO multitask RT in line with previous work (Anguera et al., 2017).

### Visuomotor Assessments

#### Visuomotor Integration

##### Beery Visuomotor Integration (VMI)

We administered the Beery Visuomotor Integration (VMI; Beery and Beery, 2004) to assess coordinated visual perception and motor coordination abilities. The Beery VMI is a pencil and paper task requiring the participant to draw copies of 30 geometric forms which increase in complexity. A trained administrator scores the copied items as either correct or incorrect based upon the criteria listed in the Beery VMI Scoring Manual (Beery and Beery, 2004). A participant can score between 0 and 30 on this assessment.

#### Visuomotor Coordination

##### Beery Visuomotor Coordination

We administered the Beery VMI Motor Coordination Subtest (Beery and Beery, 2004) to assess fine motor control abilities. The Beery motor control subtest is a pencil and paper task requiring the participant to trace the interior of 30 geometric forms which increase in complexity, without crossing over the shape’s border. A trained administrator scores the traced items as either correct or incorrect based upon the criteria listed in the Beery Motor Coordination Subtest Scoring Manual (Beery and Beery, 2004). A participant can score between 0 and 30 on this assessment.

#### Visuomotor Tracking

##### EVO—Navigation

As a part of the EVO assessment, participants complete a visuomotor tracking task requiring them to tilt the iPad to navigate their character through a dynamically moving road while avoiding walls and obstacles. Here we analyzed performance on the navigation only assessment to measure visuomotor performance without the added cognitive load of the simultaneous perceptual discrimination task (see “EVO—MULTI” section in Cognitive Control Methods).

Like EVO—Goal Management, EVO—Navigation also follows an adaptive algorithm by changing the level of steering difficulty, on a second-by-second basis, until the participant is performing at ~80% rate of accuracy (Klein, 2001; Leek, 2001; García-Pérez, 2013). The final navigation score is calculated as a function of the number of seconds it takes the EVO character to move forward a single unit—this movement is stymied by hitting walls and obstacles while steering. The EVO-Navigation score (which reflects visuomotor tracking performance) is the primary metric of interest for this study.

### Diffusion Tensor Imaging

#### DTI Acquisition

MR imaging was performed on a 3T Tim Trio scanner (Siemens, Erlangen, Germany) using a 12 channel head coil. Structural MR imaging of the brain was performed with an axial 3D magnetization prepared rapid acquisition gradient- echo (MPRAGE) T1-weighted sequence (TE = 2.98 ms, TR = 2,300 ms, TI = 900 ms, flip angle of 90°) with in-plane resolution of 1 × 1 mm on a 256 × 256 matrix and 160 1.0 mm contiguous partitions. Whole-brain diffusion imaging was performed with a multislice 2D single-shot twice-refocused spin echo echo-planar sequence with 64 diffusion-encoding directions, diffusion- weighting strength of *b* = 2,000 s/mm^2^, iPAT reduction factor of 2, TE/TR = 109/8,000 ms, NEX = 1, interleaved 2.2 mm-thick axial slices with no gap, and in-plane resolution of 2.2 mm × 2.2 mm on a 100 × 100 matrix. An additional image volume was acquired with no diffusion weighting (*b* = 0 s/mm^2^). The total diffusion acquisition time was 8.7 min. Structural MRI for all children was reviewed by PM, a board certified pediatric neuroradiologist. No structural anomalies or other clinically significant findings were identified.

#### DTI Pre-processing

The diffusion-weighted images were corrected for motion and eddy currents using FMRIB’s Linear Image Registration Tool (FLIRT1) with 12-parameter linear image registration (Jenkinson et al., 2002). All diffusion-weighted volumes were registered to the reference *b* = 0 s/mm^2^ volume. To evaluate subject movement, we calculated a scalar parameter quantifying the transformation of each diffusion volume to the reference. Children were excluded from analysis if their brain imaging had artifact and/or median relative displacement between volumes greater than 2 mm, where a volume represents a single diffusion directional measurement of the entire brain. A heteroscedastic two-sample Student’s *t*-test verified that there were no significant differences between the SPD and TDC cohort with respect to movement, during the DTI scan (*p* > 0.05). The non-brain tissue was removed using the Brain Extraction Tool (BET2). FA was calculated using FSL’s DTIFIT at every voxel, yielding an FA map for each subject. Out of the original sample of 62 children, 44 were including in the final DTI analysis.

#### Region of Interest DTI Analysis

Tract-Based Spatial Statistics (TBSS) in FSL (Smith et al., 2006) was used to co-register and skeletonize the diffusion maps for each subject in order to perform voxel-wise comparisons along the white matter skeleton. First, each subject’s FA map was non-linearly registered to each other subject’s FA map to identify the most representative FA map as a registration target. The registered maps were then averaged and skeletonized to the center of the white matter. Next, each subject’s FA data was projected onto this mean skeleton to obtain skeletonized FA maps per subject. Tract regions of interest (ROIs) were created according to The Johns Hopkins University (JHU) ICBM-DTI-81 White-Matter Labeled Atlas (Wakana et al., 2004). *A priori* ROIs were selected based upon existing literature suggesting white matter connections associated with cognitive and visuomotor control. ROI selection was restricted to the ALIC, the ATR, the CP, the PLIC, the PTR, the SCR, and the SLF. As right and left hemisphere ROI tracts were highly correlated (*r* ≥ 0.62, *p* ≤ 0.001), right and left tract FA values were averaged for each participant.

### Statistics

Analyses were performed in the R environment (R Core Team, 2013). To minimize highly influential data points, we removed values ± 3 SD away from the total mean in our behavioral assessments. Under this threshold, one individual was removed from the selective/sustained attention task and one individual was removed from the VMI task. To assess global cognitive control and visuomotor control, we generated composite scores that incorporate performance from each individual test into their functional domain. We first transformed scores from each direct assessment to z-scores (mean = 0, SD = 1 to keep scaling uniformed) using all participants’ data. Cognitive control composite scores were constructed by averaging z-scores calculated from the RT of the TOVA and EVO Goal Management Tasks. The cognitive control z-scores were reverse scored such that higher values were associated with better performance. The visuomotor composite score was constructed for each child by averaging z-scores calculated from the Beery VMI and EVO Navigation total scores. To assess for relationships between behavioral performance and DTI we constructed general linear model (GLM) regressions for each DTI tract including cohort as a factor to control for any confounding cohort effects. *P*-values for each set of regressions correlations were corrected for multiple comparisons using False Detection Rate (FDR; Benjamini and Hochberg, 1995) methods at a *p*-value threshold of *p* ≤ 0.05.

## Results

### Group Differences

To assess for cohort differences in demographics, we constructed *t*-tests for age and nonverbal IQ differences and chi-square difference tests for gender. Results demonstrated that the SPD and TDC cohorts did not significantly differ in age (*t*_(60)_ = −1.43, *p* = 0.16) nonverbal IQ (*t*_(58)_ = −1.21, *p* = 0.23), or gender (χ_(1)_ = 0.0, *p* = 1.0). Because there were no group-based demographic differences, we did not include these variables in subsequent analyses. We also constructed *t*-tests for cognitive control composite and visuomotor composite scores to test for group-based differences. Results indicated that the SPD group scored significantly lower than the TDC group in both cognitive control (*t*_(57)_ = −2.48, *p* = 0.016) and visuomotor control (*t*_(60)_ = −2.82, *p* = 0.006). Given the group differences which exist for cognitive and visuomotor control, we included cohort assignment as a covariate in all further analyses.

### Behavioral Correlations

**Figure 1:**
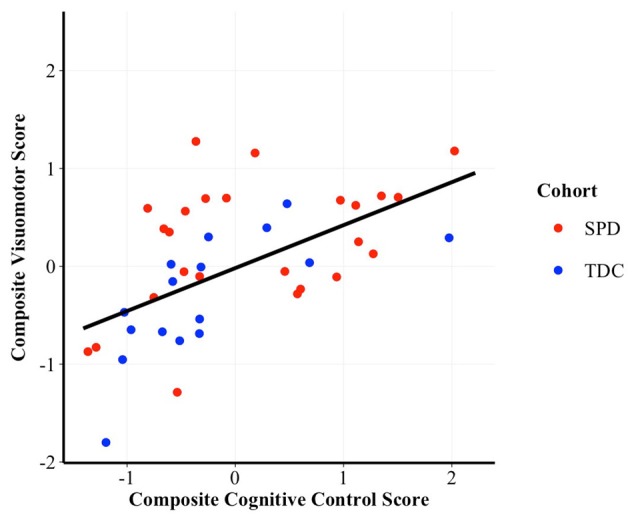
Scatterplot of the correlation between cognitive control and visuomotor control composite scores. Higher scores are indicative of better performance. *β* = 0.41, *p* = 0.001. SPD, Sensory Processing Disorder; TDC, Typically Developing Controls.

To assess whether performance on the cognitive control composite was associated with performance on the visuomotor control composite we constructed GLM regressions, controlling for cohort. Results demonstrated a significant, positive correlation between cognitive control and visuomotor control (*t*_(56)_ = 3.46, *p* = 0.001, *β* = 0.41; see Figure 1), after accounting for cohort effects.

### Neural Correlations

To assess for associations between our behavioral measure of cognitive and visuomotor control and FA values in white matter tracts of interest, we analyzed GLM regressions (controlling for cohort effects) first between composite scores (see Table 2) and secondarily within each cognitive and visuomotor task (see Table 3).

**Table 2:** Association between composite cognitive and visuomotor control tasks and white matter tract integrity.

	Cognitive control (β ± Std. Error) *N* = 42	Visuomotor control (β ± Std. Error) *N* = 44
ALIC	0.41 ± 0.14*	0.42 ± 0.13*
ATR	0.40 ± 0.14*	0.41 ± 0.13*
CP	0.19 ± 0.15	0.30 ± 0.14*
PLIC	0.17 ± 0.15	0.38 ± 0.13*
PTR	0.25 ± 0.16	0.23 ± 0.15
SCR	0.36 ± 0.15*	0.20 ± 0.15
SLF	0.40 ± 0.14*	0.38 ± 0.14*

β value regression coefficients are reported to assess for strength of the relationship after controlling for cohort assignment. ALIC, Anterior Limb of the Internal Capsule; CP, Cerebral Peduncle; PLIC, Posterior Limb of the Internal Capsule; PTR, Posterior Thalamic Radiation; SCR, Superior Corona Radiata; SLF, Superior Longitudinal Fasciculus. *Indicates FDR adjusted p-value ≤ 0.05 for regression co-efficient.

**Table 3:** Associations between individual cognitive and visuomotor control tasks and white matter tract integrity.

	Selective/sustained attention (*n* = 40)	Goal management (*n* = 39)	Visuomotor integration (*n* = 43)	Visuomotor control (*n* = 44)	Visuomotor tracking (*n* = 40)
ALIC	0.43 ± 0.15*	0.44 ± 0.15*	0.43 ± 0.14*	0.33 ± 0.15	0.09 ± 0.15
ATR	0.42 ± 0.15*	0.37 ± 0.15*	0.40 ± 0.14*	0.35 ± 0.15	0.08 ± 0.15
CP	0.24 ± 0.16	0.18 ± 0.16	0.37 ± 14*	0.15 ± 0.16	0.11 ± 0.15
PLIC	0.17 ± 0.16	0.19 ± 0.16	0.49 ± 0.13*	0.19 ± 0.15	0.11 ± 0.15
PTR	0.24 ± 0.17	0.15 ± 0.17	0.26 ± 0.16	0.28 ± 0.16	−0.06 ± 0.16
SCR	0.33 ± 0.15^∧^	0.37 ± 0.15*	0.07 ± 0.16	0.05 ± 0.16	0.31 ± 0.14
SLF	0.35 ± 0.15^∧^	0.43 ± 0.15*	0.27 ± 0.15^∧^	0.28 ± 0.15	0.21 ± 0.15

β value regression coefficients are reported to assess for strength of the relationship after controlling for cohort assignment. ALIC, Anterior Limb of the Internal Capsule; CP, Cerebral Peduncle; PLIC, Posterior Limb of the Internal Capsule; PTR, Posterior Thalamic Radiation; SCR, Superior Corona Radiata; SLF, Superior Longitudinal Fasciculus. Attention is assessed with the TOVA, goal management is assessed with EVO-MULTI, visuomotor integration is assessed with the Beery VMI, and visuomotor tracking is assessed with EVO-Nav. *Indicates FDR adjusted p-value ≤ 0.05 for regression co-efficient. ^∧^Indicates FDR adjusted p-value ≤ 0.10 for regression co-efficient.

**Figure 2:**
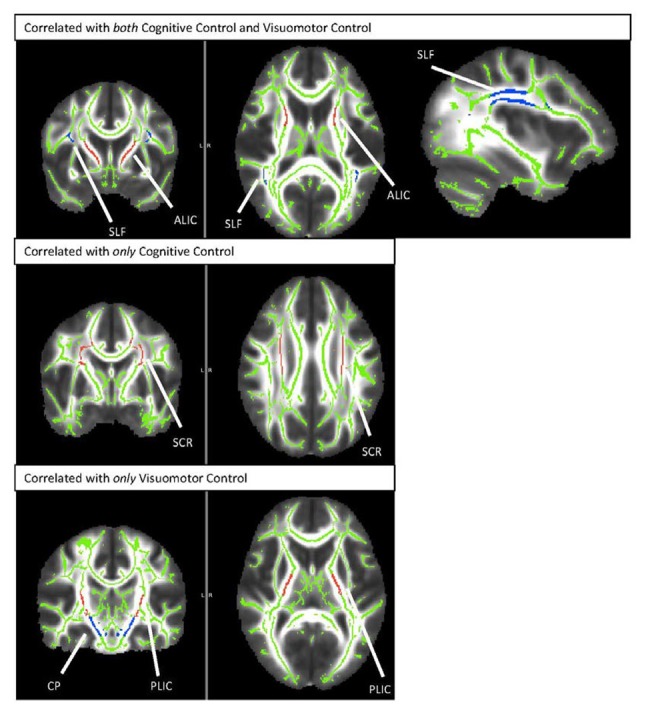
TBSS rendering of the tracts demonstrating correlations with cognitive control and/or visuomotor control. SLF, Superior Longitudinal Fasciculus; ALIC, Anterior Limb of the Internal Capsule; SCR, Superior Corona Radiata; CP, Cerebral Peduncle; PLIC, Posterior Limb of the Internal Capsule.

Results from this analysis revealed significant, positive associations between the cognitive control and visuomotor control composite scores and multiple DTI FA tracts (see Figure 2) including the anterior thalamic tracts of the ALIC and ATR and the long association fibers of the SLF. White matter microstructure of the SCR was positively associated with cognitive control performance and FA in the CP and PLIC was positively correlated with visuomotor control performance. The white matter microstructure of the PTR did not significantly correlate with either cognitive or visuomotor control composite scores.

The sustained/selective attention task (TOVA) showed significant, positive associations with FA in the ALIC and ATR and trend level positive associations with FA in the SCR and SLF. Similarly, the goal management task (EVO—MULTI) demonstrated significant positive associations with the ALIC, ATR, SCR and the SLF. In the visuomotor coordination domain, results demonstrated a significant relation between performance on the VMI task (Beery VMI) and FA in the ALIC, ATR, CP and PLIC and a trending relation with FA in the SLF. The visuomotor coordination (Beery Motor Subtest) and the visuomotor tracking task (EVO—NAV) did not demonstrate any significant brain-behavior associations.

## Discussion

This study aimed to explore the domains of cognitive and visuomotor control across children with and without SPD. The main objective of this study was to assess the existence of a common set of control-related neural tracts which may support both cognitive and visuomotor control processes. Results from this study indicate that, indeed, cognitive control and visuomotor control are behaviorally associated, and have shared and divergent neural tracts that contribute to the variability in this relationship. This finding is valuable given that both cognitive control and visuomotor control are predictive of academic readiness (Cameron et al., 2012); thus targeting these control deficits with interventions and utilizing brain based metrics for assessing change will be instrumental in ongoing work to support children with neurodevelopmental challenges.

### Cognitive Control and Visuomotor Control Associations

The outcomes from our behavioral regressions indicate that there is a relationship between cognitive and visuomotor control, such that children who struggle in one domain are more likely to struggle in the other, regardless of cohort assignment. These findings add to a growing literature positing a concordant cognitive and visuomotor control behavioral model (Diamond, 2000; Brandes-Aitken et al., 2018). Another possibility which should be considered is that cognitive control and visuomotor control are not interrelated, but that children with impaired attention and motor control are more globally impaired in all domains. However, we find this to be an unlikely explanation given that children with neurodevelopmental disorders often show unique phenotypic profiles such that a child who struggles in some domains are not necessarily burdened in all domains (Simonoff et al., 2008; Wåhlstedt et al., 2009).

### Common and Distinct Neural Elements of Cognitive and Visuomotor Control

To further substantiate the framework of a shared control system network supporting both visuomotor and cognitive control, we constructed GLM regressions controlling for cohort effects between our *a priori* DTI tracts and behavioral performance on direct assessments. These regression analyses revealed a common set of neural tracts, the ALIC, ATR, and SLF that supported performance on both cognitive control and visuomotor control composite scores. Thus, we propose that these fibers compose a putative set of control-related neural tracts, capable of supporting both cognitive control and visuomotor control.

This control network consists of two frontothalamic projection tracts, the ALIC and ATR, and a long cortical association tract, the SLF. The frontothalamic contribution to visuomotor functioning, can be interpreted from multiple perspectives. First, the ALIC and ATR largely subsume fibers to the prefrontal cortex, an area which enables us to store items in our working memory (Curtis and D’Esposito, 2003; Funahashi, 2006; Zanto et al., 2011), cognitively manipulate information (Rougier et al., 2005; Kim et al., 2011), inhibit inappropriate responses (Madsen et al., 2010; Sharp et al., 2010), and attend to relevant information (Desimone and Duncan, 1995; Schafer and Moore, 2011; Squire et al., 2013). It is plausible that visuomotor control relies upon these frontal lobe mechanisms (Diamond, 2000). Similarly, the prefrontal cortex shares neural pathways to the premotor cortex (Barbas and Pandya, 1987; Burman et al., 2014) and pre-supplementary motor area (pre-SMA; Luppino et al., 1993; Tanji, 1994), both of which are included in the frontal lobe. The pre-SMA is thought to be involved in movement preparation, coordination, and decision making (Matsuzaka et al., 1992; Nachev et al., 2008; Wilson et al., 2014) and the premotor cortex is believed to support and prepare for sensory guided movement (Passingham, 1985; Chouinard and Paus, 2006). Therefore, the inter-wirings between the prefrontal cortex, pre-SMA, and premotor cortex are likely explaining some of the overlap in behavioral challenges between cognitive and visuomotor control previously reported in children with sensory processing and neurodevelopmental challenges (Brandes-Aitken et al., 2018).

The SLF, which bridges the aforementioned frontal regions to the parietal regions, has historically predicted attention (Pavuluri et al., 2009; Cortese et al., 2013; de Luis-García et al., 2015) and visuomotor control (Langevin et al., 2014; Biotteau et al., 2016) in separate studies. Here, we demonstrate a similar effect but augment this finding in the literature by assessing both domains in the same sample of children, to more clearly compare these processes. To further understand why this set of neural tracts is associated with performance on both cognitive control and visuomotor control, it is important to consider their relationship from a neurocognitive perspective. It could be posited that the ALIC and ATR relay incoming sensory information through the thalamus to the frontal cortex (including the prefrontal, supplementary motor and premotor cortex) and from there, the SLF bridges connections to the primary motor cortex and posterior cortical regions which have historically been associated with visuomotor functioning (Steele et al., 2012; Sripada et al., 2015; Biotteau et al., 2016).

While our analyses revealed shared white matter tracts which correlated with both control processes, we also discovered some tracts which were uniquely associated with either cognitive control or visuomotor control. Specifically, our regression analysis demonstrated an association between visuomotor control and the CP, a tract which includes fibers from the corticospinal tract to the internal capsule, and the PLIC, a region which conducts sensory input from the thalamus to the posterior cortex and back to the CPs. In the context of visuomotor control, both pathways are often cited as critical to supporting coordinated motor function (Martin, 2005; Paulin, 2008; Zwicker et al., 2012a; Koziol et al., 2014; Song et al., 2015). Likewise, cognitive control performance was found to be associated with white matter microstructure in the SCR which connects the internal capsule to frontal-parietal cortical regions. Given that the cognitive control composite score incorporated multiple domains of attention and executive function, it is likely that both frontal and parietal regions support assayed performance (Sylvester et al., 2003; Collette et al., 2006). The PTR, which was included in our tracts of interest group, did not correlate with either cognitive or visuomotor control. It is possible that the lack of association suggests that although this tract is important for some aspects of sensory processing, it is not critical to cognitive or visuomotor control in children.

Within each individual task, the TOVA assessment of sustained/selective attention showed trend-level associations with FA in the ALIC and ATR, which is in line with the vast majority of literature connecting attentional control to the frontal cortex (Desimone and Duncan, 1995; Knight et al., 1995; Dimitrov et al., 2003; Sharp et al., 2010; Schafer and Moore, 2011). In addition, results demonstrated that FA in the ALIC, ATR, SCR and the SLF were associated with goal management abilities. Goal management processes draw on higher-order cognitive control and perceptual reasoning skills (Levine et al., 2000; Salthouse, 2005; Anguera and Gazzaley, 2015). Given that successful goal management depends on multiple interacting cognitive domains, it is likely supported by more wide spread cortical and sub-cortical regions including thalamo-frontal and parietal tracts (Herath et al., 2001; Nebel et al., 2005; Collette et al., 2006).

In the visuomotor domain, our results demonstrated that VMI performance was significantly associations with FA in the ALIC, ATR, the PLIC, and the CP. These findings are consistent with the literature connecting white matter integrity in the CP and PLIC to motor control output (Martin, 2005; Paulin, 2008; Koziol et al., 2014). Both the CP and PLIC are subsumed by fibers that connect the primary motor cortex with the cerebellum which is believed to support smooth motor execution (Shibasaki et al., 1993; Sanes and Donoghue, 2000; Wing, 2000; Chouinard and Paus, 2006). The significant association between VMI and white matter microstructure of the ALIC and ATR, which projects to frontal regions, suggests that the same frontal-dependent control abilities that support attention and inhibitory control may also support visuomotor control. Moreover, the close anatomical proximity and neural interconnections between the prefrontal cortex and the premotor and supplementary motor cortex highlight importance of frontal terminating white matter fibers in supporting visuomotor abilities. These findings suggest that evaluations of dyspraxia could benefit from the inclusion of cognitive control measures in addition to motor assessments to guide essential elements of remediation (Smits-Engelsman et al., 2001; Furuya et al., 2015). Surprisingly, the visuomotor coordination and visuomotor tracking assessment did not show significant correlations with the selected ROIs. The visuomotor coordination task followed similar directionality to the VMI task, but failed to reach significance. This could suggest that it is the integration aspect over the coordination component of visuomotor abilities that are supported by the selected tracts. Alternatively, given our relatively small sample size and increased variability within the visuomotor coordination task, our regressions may have been underpowered to detect true associations. Moreover, this visuomotor tracking task is substantially different from the latter two tasks and requires individuals to visually track a dynamic stimulus while integrating feedback to correct their performance. The possibility exists that these complex processes are not captured by structural white matter integrity within the tracts analyzed. Moreover, previous literature studying visuomotor tracking have primarily demonstrated associations using functional imaging methods (Brown et al., 2004; Grafton et al., 2008; Kashiwagi et al., 2009), which may capture its variability over neural architecture.

Collectively, the observed brain-behavior associations within this sample of children with rich variability in sensory, attention, and motor domains, suggest one possible theory of a unified neural network that supports and integrates sensation/perception processes with inhibition-based cognitive and visuomotor abilities. Specifically, the reported white matter microstructural findings are in line with previous research identifying a frontal cortico-thalamic circuit that is involved in both goal-directed cognitive and motor control (Jahanshahi et al., 2015). The ATR, ALIC, and SLF constitute an important part of the structural architecture within this circuit that connects the thalamus to various regions within the frontal cortex. Within this network, the striatum, thalamus and subthalamic nucleus, mediate connections from the basal ganglia (an inhibitory control center) to various regions within the frontal cortex (including prefrontal and motor regions). This unifying inhibitory-based neural framework provides a neural explanation for the concurrent overlap observed between sensory processing integration abilities and cognitive control and visuomotor control processes. Given that the sample composition within this study has allowed for optimal variation within the domains of sensory processing, cognitive control and visuomotor control, we were offered a unique opportunity to investigate the sensory integration origins of cognitive and visuomotor control overlap. While these findings offer preliminary support of this idea, future experimental research is needed to confirm whether distinct structural and functional networks underlie observed comorbid cognitive, visuomotor and sensory modulation challenges in children with neurodevelopmental disorders.

This study offers a biological explanation for why there is strong but not absolute concordance between ADHD and DCD (Rasmussen and Gillberg, 2000). Further research investigating the theory of a synchronized control system is warranted to inform future invention research. Specifically, findings from the current study offer preliminary evidence that interventions targeting visuomotor control networks could have a positive influence on cognitive control processes, and vice-versa. Existing cognitive control intervention studies have demonstrated some promising outcomes in children that extend to benefits in academic performance and intellectual abilities (Shalev et al., 2007; Jaeggi et al., 2011; Cortese et al., 2015). Similarly, visuomotor control-related intervention efforts have been explored in occupational therapy research trials with subjects showing promising improvements in multiple domains including, sensory experiences, academic achievement and motor-coordination abilities (Humphries et al., 1990; Mandich et al., 2001; Polatajko and Cantin, 2005). Furthermore, research using an integrated attention and visuomotor training program has demonstrated pronounced effects on both cognitive and visuomotor domains in children (Anguera et al., 2017) and older adults (Anguera et al., 2013). Collectively, childhood remediation efforts aimed at both cognitive and visuomotor control may have the potential to support a positive developmental trajectory.

## Limitations

While these findings offer new information to the literature, there are several limitations to this study that merit further investigation. First, we are limited by our relatively small sample size. This study would benefit by increasing our sample of children with SPD given that we could conduct regressions within each group instead of across groups which would minimize the group heterogeneity. Furthermore, many of these interpretations would be better supported following the use of temporally-locked functional neuroimaging research (e.g., EEG or fNIRS) to directly test these claims. Finally, current efforts are limited by lack of ecological validity and would be improved by including classroom-based measures of correlates of cognitive and visuomotor control (i.e., teacher reports, academic scores). Bridging this research from the lab to a school-based environment is a critical next step to inform intervention methods and benefits.

## Conclusion

In summary, the current study demonstrates behavioral overlap of cognitive and visuomotor control across children with and without sensory process dysfunction. Further, the outcomes from these results support a proposed set of common neural white matter tracts which explain the strong but not complete concordance between cognitive and visuomotor control. This work emphasizes the importance of assessing children with neurodevelopmental disorders with these overlapping control abilities in mind rather than treating diagnostic labels as standalone conditions.

## Author Contributions

AB-A contributed to the data curation, formal analysis, and writing of the manuscript. JA contributed to the study conceptualization, data curation, methodology, and writing of the manuscript. Y-SC contributed to the methodology and data processing. CD contributed to the analysis and writing of the manuscript. JO contributed to the data processing, visualization, and supervision of analysis. AG contributed to study conceptualization and methodology. PM contributed to the study conceptualization and study supervision. EM contributed to the study conceptualization, investigation, funding acquisition and writing of the manuscript.

## Conflict of Interest Statement

The authors declare that the research was conducted in the absence of any commercial or financial relationships that could be construed as a potential conflict of interest.
